# Vitiligo: Ruxolitinib and Other Oral Treatment Options Beyond Ruxolitinib

**DOI:** 10.1111/srt.70276

**Published:** 2025-10-21

**Authors:** Hira Ghani, Isabella J. Tan, Sasha Ghofrani, Madeline Tchack, Babar Rao

**Affiliations:** ^1^ Department of Dermatology The Warren Alpert Medical School of Brown University Providence Rhode Island USA; ^2^ Department of Dermatology Robert Wood Johnson Medical School–Rutgers University New Brunswick New Jersey USA; ^3^ Philadelphia College of Osteopathic Medicine Philadelphia Pennsylvania USA

**Keywords:** autoimmune, JAK inhibitors, phototherapy, repigmentation, systemic treatments, vitiligo

## Abstract

**Background:**

Vitiligo is a chronic autoimmune disorder marked by the loss of melanocytes, causing depigmented patches across the skin. Affecting up to 2% of the global population, vitiligo presents significant psychological impacts, particularly in those with darker skin types. Current hypotheses attribute its etiology to autoimmune destruction, genetic predisposition, and environmental triggers like trauma and stress.

**Materials and Methods:**

A comprehensive review of recent studies was conducted using PubMed and Google Scholar, focusing on systemic treatments for vitiligo, particularly JAK inhibitors (ruxolitinib, upadacitinib, tofacitinib, ritlecitinib, and baricitinib). Eligible studies included trials and case reports on adults with vitiligo evaluating treatment efficacy, safety, and tolerability.

**Results:**

Topical ruxolitinib has achieved the highest efficacy in repigmenting facial and sun‐exposed areas, particularly when combined with phototherapy. Emerging evidence supports oral Janus kinase (JAK) inhibitors, especially upadacitinib and tofacitinib, for their repigmenting effects in refractory cases, though side effects like acne and neutropenia warrant monitoring. Limited evidence supports methotrexate and minocycline as adjunct therapies, with minor repigmentation observed.

**Conclusion:**

Advances in systemic and topical JAK inhibitors have shown promising repigmentation effects in vitiligo, particularly for localized facial lesions. Ruxolitinib cream, the first FDA‐approved therapy, and other JAK inhibitors highlight a growing therapeutic approach, with ongoing trials needed to optimize efficacy and evaluate long‐term safety. Combination therapy with phototherapy shows enhanced repigmentation outcomes.

## 1 Introduction

Vitiligo is an acquired, chronic skin disorder characterized by the loss of functional melanocytes, resulting in depigmented macules and patches on the skin [1]. Affecting approximately 0.5%–2% of the global population, vitiligo presents across all ethnicities and age groups, though its psychological and social impact is often more profound in individuals with darker skin types [2]. The disease has no gender preference and commonly manifests before the age of 20 in nearly half of patients [3].

The pathophysiology of vitiligo is complex, involving both genetic predisposition and environmental factors [4]. Autoimmune destruction of melanocytes is considered the central mechanism, supported by the presence of melanocyte‐specific autoantibodies and T‐cell infiltration in lesional skin [5]. The disease is also associated with oxidative stress, as studies have shown elevated levels of reactive oxygen species in the skin of vitiligo patients, further contributing to melanocyteapoptosis [6]. Genetic studies have shown that vitiligo susceptibility genes largely encode immune and melanocyte proteins, linking vitiligo to melanoma and natural variations in pigmentation [7].

Several hypotheses have been proposed to explain vitiligo's etiology, including the autoimmune, neural, and biochemical models [8]. The autoimmune hypothesis remains dominant, based on evidence of autoantibodies targeting melanocytes and the frequent association of vitiligo with other autoimmune disorders, such as autoimmune thyroiditis and type 1 diabetes [9]. Environmental triggers such as trauma (Koebner phenomenon), stress, and sunburn are also implicated in the disease's onset or exacerbation [10].

Management of vitiligo remains challenging, though several therapies have emerged to slow progression and induce repigmentation (Table 1). Topical corticosteroids and calcineurin inhibitors are typically first‐line for early‐stage or localized disease [11]. Targeted phototherapy—including narrowband ultraviolet B (NB‐UVB), excimer laser, and excimer lamp—should be considered the backbone of treatment and is often employed for more extensive or refractory cases, particularly when >5%–10% of the body surface area (BSA) is affected [12]. The topical Janus kinase (JAK) inhibitor ruxolitinib, the first FDA‐approved treatment for repigmenting non‐segmental vitiligo in patients ≥12 years with ≤10% BSA involvement, has shown promise through modulation of immune signaling pathways [13]. A detailed clinical history and laboratory screening should be performed prior to initiating JAK inhibitors to assess for contraindications and ensure patient safety. Systemic therapies, including oral corticosteroids and investigational oral JAK inhibitors, are being explored for rapidly progressing or widespread disease [14]. This review examines recent literature from the past 5 years evaluating the efficacy and tolerability of both established and emerging topical and oral treatments for vitiligo, with a particular focus on JAK inhibitors.

**TABLE 1:** Comparison of vitiligo treatments.

Drug	Mechanism	Research designs	Overall efficacy	Percentage of repigmentation	Adverse effects	Recommendation
**Ruxolitinib *topical*** [13, 15, 16, 17]	JAK 1/2 inhibitor	Phase II RCT, Phase III RCT, Open‐label trial, Review articles	Significant repigmentation, greater epidermal/dermal concentration in topical	**VASI**: 23% at 20 weeks **F‐VASI**: 50%–90% dose‐dependently	Application site acne, rash, and pruritus	Twice‐daily topical use
**Ruxolitinib *oral*** [15, 16, 17]	JAK 1/2 inhibitor	Case reports	Limited studies, reports of re‐pigmentation	N/A	Consider systemic adverse effects of immunosuppressant	Further research needed
**Upadacitinib** [18, 19, 20, 21]	JAK1 inhibitor	Phase II RCT, Research letter, Prospective case series, Case study	Significant repigmentation	**VASI**: 38.6% **FVASI**: 51.4% **Case study**: 90% face/neck, 60% chest	Minor reactions: acne, headache, fatigue, COVID‐19	Significance with 11, 15, and 22 mg doses. Insignificant with 6 mg dose
**Tofacitinib** [17, 22, 23, 24]	JAK1/3 inhibitor	Case studies, Retrospective case series	Complete or near complete repigmentation	95% facial/scalp improvement by 11 months [17]	No adverse reactions reported	5‐10 mg doses successful. Adjunctive phototherapy
**Ritlecitinib** [25, 26, 27, 28]	JAK 3 and TEC inhibitor	Phase IIb RCTs, Post hoc analysis	Significant repigmentation, faster on darker skin and stable lesions	N/A	No adverse reactions reported	50 and 30 mg + loading dose successful. Accelerated improvement with 50 mg + loading dose
**Baricitinib** [29, 30, 31, 32, 33]	JAK 1/2 inhibitor	Case studies, Case series, Prospective open‐label study, Meta‐analysis	Significant repigmentation with concurrent phototherapy	**F‐VASI75** achieved in 93.3% (*n* = 17, with NB‐UVB); **T‐VASI50** achieved in 70.6% at 16 weeks; **Case reports**: >75% repigmentation by 6–8 months; 25.9% average lesion reduction by 5 months	Most studies had no common adverse reactions	Adjunctive phototherapy
**Oral corticosteroids** [34]	Immunosuppressive, anti‐inflammatory	RCT	Stabilization, partial repigmentation	N/A	Weight gain, hypertension, osteoporosis	Short‐term pulse therapy
**Minocycline** [35, 36]	Anti‐inflammatory, antioxidant	RCT	Modest repigmentation; no synergy with NBUVB	**Mean VASI reduction**: 28.9% (NBUVB + minocycline) vs. 27.3% (NBUVB alone) no significant difference	Hyperpigmentation (localized)	Investigational adjunct
**Methotrexate** [37, 38]	Immunosuppressive	Case series, Observational studies	Disease stabilization; occasional repigmentation	N/A	Hepatotoxicity, GI issues, marrow suppression	Refractory and progressive cases

Abbreviation: RCT, randomized controlled trial.

## 2 Methods

A comprehensive literature search was conducted to identify studies evaluating oral treatment options for vitiligo beyond ruxolitinib. The search was performed using the PubMed and Google Scholar databases, covering the past 5 years. The search terms included combinations of “vitiligo,” “oral treatments,” “systemic therapy,” “JAK inhibitors,” “ruxolitinib,” “upadacitinib,” “tofacitinib,” “baricitinib,” “ritlecitinib,” “immunomodulators,” and “systemic immunotherapy.” References of relevant articles were also screened.

Eligible articles included clinical trials, cohort studies, case reports, and reviews focused on the efficacy, safety, and tolerability of oral treatments for vitiligo. Studies were restricted to those published in English and included adult populations with vitiligo. Articles focused on topical therapies or non‐systemic treatments were excluded. Data extraction focused on treatment type, study design, sample size, outcomes measured (e.g., repigmentation, disease stability), and adverse effects.

## 3 Discussion

### 3.1 JAK Inhibitors

#### 3.1.1 Ruxolitinib

##### 3.1.1.1 Topical

Ruxolitinib is a topical JAK 1/2 inhibitor that has been used as a topical treatment for vitiligo [15]. A phase II open‐label trial studying the use of twice‐daily topical ruxolitinib in treating vitiligo found 23% improvement in Vitiligo Area Scoring Index (VASI) score at week 20 (*p* = 0.02). The most significant response was repigmentation of the face (*p* = 0.001), including one patient with 76% facial improvement [15, 16]. A phase II randomized, double‐blind study selected 157 patients with vitiligo and assigned them to different doses of topical ruxolitinib (once daily of 0.15%, 0.5%, 1.5%, and 1.5% twice daily) or control group. Patients treated with ruxolitinib were found to have Facial VASI (FVASI) scores of >50%, >75%, and >90%. Facial scores had higher rates with hand and foot involvement with lowest response rates. Total VASI scores over 50% had a dose‐dependent relationship [15, 16, 17]. In two phase III double‐blind vehicle‐controlled trials, 674 patients were treated with 1.5% ruxolitinib cream twice daily for 24 weeks then an additional 28 weeks [13, 15]. At 24 weeks, an average of 30% of treated patients showed an F‐VASI score greater than 75. At 52 weeks, there was an overall significant repigmentation compared to control. Adverse reactions included application site acne, rash and pruritus, as well as nasopharyngitis, headache, pyrexia, and few with application site exfoliation [13, 15]. With ongoing trials, topical ruxolitinib is still being researched in its treatment of vitiligo.

### 3.2 Oral

In terms of oral versus topical ruxolitinib, topical has been more commonly used. Some studies have found that topical has a greater epidermal and dermal concentration than oral [15, 17]. There have only been a few cases of oral ruxolitinib used in vitiligo causing repigmentation; however, depigmentation arose once medications were stopped [15, 17]. With systemic administration, thrombocytopenia, neutropenia, blood clots, major heart issues, and anemia are adverse reactions to consider [16]. Research is limited and further studies would be needed to establish the safety and efficacy of oral ruxolitinib in vitiligo repigmentation.

### 3.3 Upadacitinib

Upadacitinib is an oral selective JAK1 inhibitor that is currently being researched for its efficacy in treating vitiligo [18]. A study of 12 patients with refractory vitiligo researched safety and efficacy of treatment with oral upadacitinib [18]. A 15 mg daily dose was given for 16 weeks. At follow up, there was an average improvement of 38.6% (0%–90%). Of the 12 patients, five developed 50% improvement in VASI scores. Facial‐VASI (FVASI) average improvement was 51.4% in seven patients, which was a greater percentage than acral lesions (16.8% improvement). Dermatology Life Quality Index (DLQI) score developed 41.9% average enhancement [18]. In a prospective case series, 5 of 15 patients received upadacitinib, and took 15 mg daily for 6 months [19]. VASI scores went from a 3‐month average of 16.77.44 ± 9.73 to a 6‐month average of 3.02 ± 17.08 (*p* = 0.004). In terms of adverse effects, 10% reported adverse events experienced acne, with no severe reports [19]. In a phase II multicenter, randomized, double‐blind clinical trial, 185 patients with non‐segmental vitiligo were randomly assigned multiple doses of upadacitinib and assessed for improvement of VASI scores [20]. Doses included 6 , 11, 22 mg, or placebo. Results showed a significant improvement in repigmentation to facial and total body vitiligo lesions, especially at 11 and 22 mg doses. *p* values at 6 mg were insignificant (*p* = 0.3, 0.12). The most common adverse effects were COVID‐19, headache, fatigue, and acne [20]. Further research into oral upadacitinib includes a case study of one 16‐year‐old male patient with atopic dermatitis and rapidly progressive vitiligo treated with 15 mg upadacitinib and crisaborole [21]. At 4 months, face and neck repigmentation was at 90%, chest repigmentation at 60%, and extremities showed minimal improvement. However, concurrent use of crisaborole is important to consider as a variable. Adverse reactions included acne worsening. Dose was decreased by half after 4 months, with no recurrence after 3 months [21]. Overall, upadacitinib has had promising results, especially with facial repigmentation, and has shown minor adverse reactions where it is now being moved into phase III trials [20].

### 3.4 Tofacitinib

Tofacitinib is an oral JAK1/3 inhibitor available in both oral and topical forms. In a case study using 5 mg daily oral tofacitinib, a patient with alopecia areata and vitiligo achieved successful repigmentation after 5 months of treatment, with only 5% of depigmented macules remaining [17]. Another retrospective study of 10 patients who took 5–10 mg daily for around 10 months found that half experienced repigmentation in sun‐exposed areas or areas treated with phototherapy [17]. These findings suggest that JAK inhibitors combined with light exposure may improve repigmentation [17]. In a separate case study, a male patient with vitiligo received 5 mg oral tofacitinib twice daily [22]. He began with 70% depigmentation of BSA, and by 11 months, he had nearly complete repigmentation of the face and scalp, with the most improvement in sun‐exposed areas. After 5 years, there has been continued gradual repigmentation in all areas except the axillary and inguinal folds [22]. Two case reports of female patients with vitiligo showed marked improvement or full resolution of vitiligo lesions with 5 mg twice daily [23, 24]. No adverse reactions were reported in any of these cases [17, 22, 23, 24]. However, one case study reported new‐onset vitiligo in a patient taking tofacitinib for rheumatoid arthritis, suggesting possible paradoxical effects that warrant further investigation [39]. Overall, tofacitinib has shown promise for successful repigmentation in vitiligo patients, especially in facial and sun‐exposed areas, though further research is needed to better understand its efficacy.

### 3.5 Ritlecitinib

Ritlecitinib, an irreversible JAK 3 and TEC inhibitor, is a novel drug being explored for vitiligo treatment [40]. In a randomized phase 2b clinical trial involving 364 patients treated with varying dosages of oral ritlecitinib, a significant difference in F‐VASI scores was observed in the 50 mg group (*p* < 0.001) and in the group receiving 30 mg without a loading dose (*p* = 0.01) [40]. Accelerated improvement was noted in the group with a 200 mg loading dose followed by a 50 mg maintenance dose. No adverse events were reported over the 48‐week treatment period [40]. A follow‐up post hoc analysis of this study examined efficacy across Fitzpatrick skin types and found that participants with darker skin responded faster than those with lighter skin, due to differences in immune biomarkers [25]. Additionally, stable lesions exhibited greater and earlier repigmentation than active lesions, which required decreased inflammation prior to repigmentation [26]. Another randomized, double‐blind phase 2b trial assessed ritlecitinib's effects on skin and blood biomarkers in 65 patients with non‐segmental vitiligo [41]. Immune biomarkers significantly decreased, and melanocyte‐related markers significantly increased by 4 and 24 weeks, compared to control groups (both *p* < 0.05). Changes in immune biomarkers correlated with clinical improvement in patients [41]. Due to the significant improvements observed in phase 2 trials, phase 3 trials are currently ongoing [27].

### 3.6 Baricitinib

Baricitinib, a selective JAK 1/2 inhibitor, is also currently being researched as a treatment for vitiligo. In one case study, a male patient with vitiligo on his upper extremities achieved complete repigmentation with 4 mg of baricitinib after switching from 5 mg tofacitinib [28]. In one study using oral baricitinib, the in vivo section included four patients with progressing vitiligo [29]. After 12 weeks, patients experienced significant repigmentation, though two had depigmentation by the 3‐month follow‐up. Two patients in a case report received baricitinib with narrowband ultraviolet B (NB‐UVB) phototherapy [30]. The first patient showed significant repigmentation by 8 months, while the second patient, after vitiligo stabilization with steroid use, had >75% repigmentation after 6 months [30]. A case series of five patients with vitiligo were evaluated when taking oral baricitinib with concurrent heliotherapy [31]. After 5 months, vitiligo lesions decreased by 25.9%. Repigmentation was most efficacious in sun‐exposed areas, except high friction areas including distal extremities [31]. Lastly, a recent prospective, controlled, open‐label study evaluated 17 patients taking low‐dose baricitinib with concurrent NB‐UVB. By 16 weeks, 70.6% of treated patients achieved T‐VASI50 (*p* = 0.001), and 93.3% achieved F‐VASI75 (*p* = 0.021). Minor adverse reactions reported included erythema, acne, and mild phototherapy‐induced blisters [32]. However, most studies reported no common adverse effects [28, 29, 30].

Overall, JAK inhibitors have been found to have successful treatments. A meta‐analysis of nine studies with a total of 45 patients assessed the overall efficacy of JAK inhibitors in treating vitiligo [33]. They found that 57.8% had a good response, 22.2% had partial response and 20% had no or minimal response. Facial vitiligo had the greatest response at 70% (*p* < 0.001) and the non sun‐exposed areas had the lowest response. Adjunctive phototherapy had a significantly improved efficacy [33]. When comparing JAK inhibitors, a study assessing baricitinib, upadacitinib, and tofacitinib found overall improvement in vitiligo repigmentation but no differences in efficacy between the drugs, highlighting the need for further research [19].

### 3.7 Other Oral Treatments

Beyond JAK inhibitors, additional oral treatments for vitiligo have been investigated for their immunomodulatory effects and potential to slow disease progression or induce repigmentation. These therapies, though often with varied mechanisms of action, target inflammation (i.e. oral corticosteroids, minocycline) and immune dysregulation (i.e. methotrexate), which are central to vitiligo pathogenesis [14]. Currently, evidence for the efficacy of these treatments remains limited, with most findings from smaller cohort studies, case series, and clinical experience. Further research, particularly randomized controlled trials, is necessary to establish standardized dosing and assess long‐term outcomes for these therapies.

### 3.8 Oral Corticosteroids

Oral corticosteroids are occasionally employed as short‐term pulse therapies to stabilize rapidly progressing vitiligo [14]. They act by dampening the immune response and reducing inflammation, thereby limiting further melanocyte destruction [14]. Low‐dose oral steroids such as prednisolone, given as minipulses, have demonstrated efficacy in halting the progression of active vitiligo in some cases. One randomized trial indicated that oral steroid mini pulses may achieve disease stabilization and induce partial repigmentation in certain patients, particularly in early or rapidly progressing vitiligo [34]. However, prolonged steroid use is associated with adverse effects, including weight gain, hypertension, and osteoporosis, making long‐term therapy undesirable [34]. Steroid therapy is typically limited to short‐term use or reserved for cases unresponsive to other treatments.

### 3.9 Minocycline

Minocycline, a tetracycline antibiotic with anti‐inflammatory and antioxidant properties, has been explored as an adjunct treatment for vitiligo. Its proposed mechanism involves reducing oxidative stress and modulating the immune response, thus potentially protecting melanocytes from immune‐mediated destruction [35].

Combining oral minocycline and narrowband ultraviolet B (NBUVB) phototherapy for treating generalized vitiligo has limited evidence, though each treatment individually has shown potential benefits [36]. In one randomized, double‐blind, placebo‐controlled pilot study, combination therapy with NBUVB and minocycline resulted in a mean Vitiligo Area Scoring Index (VASI) score reduction of 28.87% (SD = 24.15) compared to 27.26% (SD = 7.98) in the NBUVB‐only group, with no statistically significant difference (*p* = 0.886) [36]. Additionally, no significant differences were observed in the quartile grading scale (QGS) of repigmentation or in changes to the Vitiligo Disease Activity Index (VIDA) score [36]. Notably, 29% (two of seven) of patients in the minocycline group developed localized hyperpigmentation, suggesting that while minocycline shows promise individually, it does not enhance NBUVB efficacy in combination and may increase the risk of adverse pigmentation effects [36].

Other small studies and case reports have noted repigmentation in patients treated with minocycline, though its effect appears modest. Due to limited evidence, minocycline's role in vitiligo management remains investigational, with further trials needed to clarify its efficacy and safety profile in this patient population.

### 3.10 Methotrexate

Methotrexate, an immunosuppressive agent, has been utilized off‐label in vitiligo treatment, particularly for patients with progressive or refractory disease [37]. Acting by inhibiting folate metabolism and modulating immune responses, methotrexate may suppress the autoimmune processes that contribute to melanocyte destruction in vitiligo [37]. Some case series and observational studies have suggested that low‐dose methotrexate may stabilize disease activity and, in some instances, support repigmentation [38]. However, methotrexate's side effect profile—including hepatotoxicity, gastrointestinal distress, and risk of bone marrow suppression—necessitates careful patient selection and monitoring [38]. Although promising as an adjunct therapy, more robust studies are needed to determine methotrexate's efficacy and optimal dosing in vitiligo treatment.

## 4 Conclusion

Vitiligo remains a challenging autoimmune skin disorder characterized by melanocyte loss and notable psychological impacts, particularly for individuals with darker skin. Recent advances in treatment, particularly with JAK inhibitors like ruxolitinib, upadacitinib, tofacitinib, ritlecitinib, and baricitinib, have shown encouraging results in promoting repigmentation, especially in facial and sun‐exposed areas. Only FDA‐approved topical JAK inhibitors, such as ruxolitinib cream, have achieved notable efficacy in early trials, while systemic JAK inhibitors are being explored for broader use, albeit with potential adverse effects. Adjunct therapies like NB‐UVB phototherapy can further enhance treatment outcomes, supporting a combination approach. Ongoing research and clinical trials are essential to refine dosing, assess long‐term effects and validate these promising therapies across diverse vitiligo populations.

## Conflicts of Interest

The authors declare no conflicts of interest.

No data from this study has been previously published.

## Data Availability

The data that supports the findings of this review are available from publicly accessible sources and repositories. All references and citations to the primary studies, datasets, and literature sources utilized in this review are listed in the reference section.
